# Supplementary material for: Elevated Dipeptidyl Peptidase IV (DPP-IV) Activity in Plasma from Patients with Various Lysosomal Diseases
Source: Diagnostics (Basel). 2021 Feb 16;11(2):320. doi: 10.3390/diagnostics11020320 (PMC7920438; doi:10.3390/diagnostics11020320)
Supplement: Supplementary file 1 [file diagnostics-11-00320-s001.pdf]

diagnostics

MDPI

Article

Elevated Dipeptidyl Peptidase IV (DPP-IV) Activity in Plasma from Patients with Various Lysosomal Diseases

Agnieszka Ługowska [1,*], Galina Baydakova [2], Alex Ilyushkina [2], Ekaterina Zakharova [2], Hanna Mierzewska [3], Krystyna Szymańska [4], Jolanta Wierzba [5], Jolanta Kubalska [1], Ałła Graban [6], Tomasz Kmieć [7], Barbara Perkowska-Sumiła [8], Anna Tylki-Szymańska [8] and Małgorzata Bednarska-Makaruk [1]

1 Department of Genetics, Institute of Psychiatry and Neurology, 02-957 Warsaw, Poland; kubalska@ipin.edu.pl (J.K.); makaruk@ipin.edu.pl (M.B.-M.)
2 Federal State Budgetary Institution, Research Centre for Medical Genetics, 115478 Moscow, Russia; baydakovag@gmail.com (G.B.); alexilyuskina@gmail.com (A.I.); doctor.zakharova@gmail.com (E.Z.)
3 Department of Child and Adolescent Neurology, Institute of Mother and Child, 01-211 Warsaw, Poland; h.mierzewska@gmail.com
4 Department of Experimental and Clinical Neuropathology, Mossakowski Medical Research Center, Polish Academy of Sciences, 02-106 Warsaw, Poland; szymanska2@wp.pl
5 Department of Internal and Pediatric Nursing, Institute of Nursing and Midwifery, Medical University of Gdańsk, 80-210 Gdańsk, Poland; jolanta.wierzba@gumed.edu.pl
6 1st Department of Neurology, Institute of Psychiatry and Neurology, 02-957 Warsaw, Poland; graban@ipin.edu.pl
7 Department of Neurology and Epileptology, The Children's Memorial Health Institute, 04-730 Warsaw, Poland; t.kmiec@czd.pl
8 Department of Pediatrics, Nutrition and Metabolic Diseases, The Children's Memorial Health Institute, 04-730 Warsaw, Poland; b.perkowska@ipczd.pl (B.P.-S.); atylki@op.pl (A.T.-S.)
* Correspondence: alugipin@yahoo.com; Tel.: +48-22-4582-610; Fax: +48-22-8589169

Citation: Ługowska, A.; Baydakova, G.; Ilyushkina, A.; Zakharova, E.; Mierzewska, H.; Szymańska, K.; Wierzba, J.; Kubalska, J.; Graban, A.; Kmieć, T.; et al. Elevated Dipeptidyl Peptidase IV (DPP-IV) Activity in Plasma from Patients with Various Lysosomal Diseases. *Diagnostics* **2021**, *11*, 320. https://doi.org/10.3390/diagnostics11020320

Received: 28 December 2020
Accepted: 13 February 2021
Published: 16 February 2021

Publisher's Note: MDPI stays neutral with regard to jurisdictional claims in published maps and institutional affiliations.

Copyright: © 2021 by the authors. Licensee MDPI, Basel, Switzerland. This article is an open access article distributed under the terms and conditions of the Creative Commons Attribution (CC BY) license (http://creativecommons.org/licenses/by/4.0/).

Abstract: Increased activity of dipeptidyl peptidase IV (DPP-IV) was reported earlier in patients with different types of mucopolysaccharidoses. DPP-IV (also known as CD26 lymphocyte T surface antigen) is a transmembrane protein showing protease activity. This enzyme displays various functions in the organism and plays an important role in multiple processes like glucose metabolism, nociception, cell-adhesion, psychoneuroendocrine regulation, immune response and cardiovascular adaptation. In order to evaluate DPP-IV in lysosomal storage diseases (LSD), we examined its activity in plasma samples from 307 patients affected with 24 different LSDs and in 75 control persons. Our results revealed elevated DPP-IV activity especially in individuals affected with mucolipidosis II/III, alpha-mannosidosis, and mucopolysaccharidoses types III, II, and I ($p < 0.05$). In other LSDs the DPP-IV activity was still significantly increased, but to a lesser extent. In patients with Gaucher disease, ceroid lipofuscinosis type 1 (CLN1), Niemann–Pick disease type C and A, Krabbe and Pompe diseases, gangliosidosis GM2 and metachromatic leukodystrophy discreet or no changes in DPP-IV activity were observed. DPP-IV may serve as a first-tier diagnostic procedure or additional biochemical analysis in recognizing patients with some LSDs. DPP-IV may become an object of basic research for a better understanding of LSDs.

Keywords: DPP-IV; lysosomal diseases; mucolipidosis II/III; mucopolysaccharidoses; alpha-mannosidosis; diagnosis; screening

1. Introduction

Lysosomal storage diseases (LSDs) belong to a group of inherited metabolic disorders with a frequency ranging from about 1 in 4000 to 1 in 13,000 live births [1–4]. Among them, several groups of diseases are distinguished according to the character of stored

substrates, the most prominent of which are: sphingolipidoses, glycoproteinoses, mucopolysaccharidoses, ceroid lipofuscinoses, disorders of lysosomal membrane transport, mucolipidoses, and others. The clinical picture of LSDs is heterogeneous, including nervous, visceral, skeletal, cardiologic, gastric, dermatologic, and other signs and symptoms.

Diagnostics of LSD depends on a lacking functional protein—enzymatic, membrane or structural—and involves an analysis of enzymatic activities or the presence/absence of a functional non-enzymatic protein, analysis of stored material or detection of specific biomarkers. Due to the fact that some LSDs are treated with enzyme replacement therapy (ERT), substrate reduction therapy (SRT), pharmacological chaperones or hematopoietic stem cell transplantation (HSCT), there is a special need for quick diagnostic procedure and a tool for monitoring of LSD therapy. In this aspect, biomarkers seem to play an important role, since they enable neonatal or selective screening, can serve as an additional diagnostic parameter or can be used for monitoring of treatment efficacy.

The most popular biomarker in screening for LSD is chitotriosidase, whose activity is elevated significantly in patients with Gaucher disease and to a lesser extent also in patients suffering from Niemann–Pick disease type A/B or C, gangliosidosis GM1 and Krabbe disease [5]. Other frequently used biomarkers include lysolipids (lyso-Gb3, lyso-Gl1, lyso-SM and lyso-SM509), which are analysed in patients with a suspicion of Fabry disease, Gaucher disease or Niemann–Pick type A/B or C diseases [6–10]. Oxysterols and derivatives of bile acids are also estimated while screening for Niemann–Pick type C disease. In the case of mucopolysaccharidoses stored glycosaminoglycans (GAGs) serve as biochemical biomarkers for these disorders. There are additional other rarely used biomarkers of LSD; for a review see [11,12].

Beesley et al. reported elevated activity of dipeptidyl peptidase IV (DPP-IV) in patients with different types of MPS and suggested it be used as a biomarker for MPS screening tests [13]. This observation was further worked out by Kurt et al. [14] and Hetmańczyk et al. [15], who confirmed that DPP-IV discriminates between patients with MPS and healthy control persons.

DPP-IV (also known as CD26 lymphocyte T surface antigen) is a transmembrane protein showing protease activity; namely, it recognizes proline (Pro) or alanine (Ala)-containing polypeptides and cleaves Thr-Pro dipeptides from the N-terminus end of the peptide chain [16]. DPP-IV displays various functions in the organism and plays an important role in multiple processes like glucose metabolism, nociception, cell-adhesion, psychoneuroendocrine regulation, immune response and cardiovascular adaptation [17].

The aim of this multicenter study was to evaluate DPP-IV activity in patients with various forms of MPS and with other lysosomal diseases.

Our principal conclusion is that DPP-IV activity is elevated in patients with mucolipidosis II/III, alpha-mannosidosis and some types of mucopolysaccharidoses.

2. Materials and Methods

2.1. Patients

We obtained plasma samples from 307 patients affected with 24 different lysosomal diseases (LSD) (see Supplementary Table S1). LSD patients included in this study were all diagnosed biochemically and, in some cases, molecularly. The Warsaw control group consisted of 17 persons directed to the Department of Genetics, Institute of Psychiatry and Neurology in Warsaw, amongst whom no lysosomal disease was recognized. In the Federal State Budgetary Institution, Research Centre for Medical Genetics in Moscow, the control group consisted of 58 healthy individuals (for details, please see Supplementary Table S1).

No individual from the control groups was affected with diabetes mellitus.

The plasma samples obtained from heparinized blood were stored at −20 °C before analysis. The time between collection and assay ranged from 2 weeks to about 24 months in the Warsaw Lab and from 2 months to 3 years in the Moscow Lab. Sample storage conditions were the same in both laboratories and between samples. Samples were thawed no more than three times before performing the analysis.

The plasma samples used in this study were obtained from blood samples submitted to the Department of Genetics, Institute of Psychiatry and Neurology in Warsaw, and to the Research Centre for Medical Genetics in Moscow for routine LSD screening and other diagnostic procedures. Patients or their caregivers gave their informed consent to include their anonymized biological material in the scientific studies.

Plasma samples, surplus to the requirements for diagnosis, were collected with informed consent during the diagnostic procedures at the Institute of Psychiatry and Neurology, Department of Genetics (Warsaw, Poland), and at the Federal State Budgetary Institution, Research Centre for Medical Genetics (Moscow, Russia). Individual patient consent to participate was not sought since all procedures were in accordance with routine patient care for LSD screening and other diagnostic procedures. All experiments were performed in accordance with relevant guidelines and regulations. The study was conducted in accordance with the Declaration of Helsinki.

This article does not contain any results obtained from studies on animals.

2.2. DPP-IV Enzyme Assay

Plasma DPP-IV was analyzed using a chromogenic substrate Gly-Pro-p-nitroanilide (Sigma-Aldrich, USA, cat. no. G0513) according to the producer's protocol included in the DPPIV/CD26 Assay Kit for Biological Samples (Enzo Life Sciences). Absorbance was measured at 405 nm by a microplate reader (BioRad iMark Microplate Absorbance Reader) or on fluorometer LS55 Luminescence Spectrometer (Perkin Elmer, UK) and the amount of the released p-nitroaniline was calculated from a standard curve. The DPP-IV activity was expressed as nmol/ml/hr.

2.3. Statistical Analysis

Statistical analysis was performed using Statistica version 13.1 (StatSoft) and GraphPad Prism 6.

Multiples of the Median (MoMs) was used as a method to normalize data from participating laboratories so that individual test results could be compared. For each patient, the DPP-IV activity (absolute result) was converted to Multiple of the Median for the control group of healthy individuals. Each participating laboratory used its own controls. The use of Multiples of the Median helps to overcome analytic differences among laboratories.

Variables, i.e., DPP-IV activity (absolute result) and DPP-IV MoMs, were presented as medians with interquartile ranges (IQR). The nonparametric Mann–Whitney test was used to evaluate statistical differences between the investigated groups and controls. p-value < 0.05 was considered statistically significant.

Receiver operating characteristic (ROC) curve analysis was used to assess the usefulness of DPP-IV activity as a biomarker for classifying disease status and allowed to define the cut-off values for both the DPP-IV activity, as well as the DPP-IV MoMs for all groups of patients with various lysosomal diseases and controls. The area under the ROC curve (AUC) is considered as an effective measure of the validity of a diagnostic test. The larger the AUC, the better the overall performance of the test in correctly identifying diseased and non-diseased subjects. The maximal Youden index was used to find the optimal cut-off point to least misclassify diseased and non-diseased subjects.

3. Results

Figure 1, Table 1, Supplementary Figure S1 and Supplementary Table S2 show the median and IQR and minimum–maximum range of DPP-IV activity (crude data) and DPP-IV activity expressed as MoM (Multiple of the Median) of the control group in 24 various lysosomal diseases.

In order to present results obtained in our two laboratories in a reliable, objective manner, we compared the multiples (folds) of medians of DPP-IV activity characteristic of patients with different LSDs and control individuals (MoM). In cases where the group of patients was not numerous, only its mean was compared to the median of the control group. Very high DPP-IV activity was found in patients with mucolipidosis type II/III (over 5 MoM or 20 MoM in Warsaw or Moscow, respectively), alpha-mannosidosis (4.87 MoM, Warsaw) and mucopolysaccharidosis type II (6.06 MoM in Moscow and 3.16 MoM in Warsaw). High DPP-IV activity, over 3 MoM was observed in individuals affected with gangliosidosis GM1 and in two patients with Niemann–Pick type B (n = 1) and mucopolysaccharidosis type IIIC (n = 1), see Table 1 and Supplementary Table S2.

Elevated DPP-IV activity, between 1.75 and 7 MoM was detected in patients with other MPS, sialidosis, Wolman/CESD, MSD and CLN2 (in this case the MoM in the Moscow Lab was as high as 5.37). It is worth noting that the results obtained in the Moscow Laboratory were higher than in the Warsaw Lab.

Slightly changed or unchanged DPP-IV activity was detected in patients with Gaucher disease (not treated), ceroid lipofuscinosis type 1 (CLN1; n = 1), Niemann–Pick type C and A, Krabbe, Pompe diseases (not treated), gangliosidosis GM2 and metachromatic leukodystrophy (MLD). In patients with Fabry disease (not treated) DPP-IV activity was mostly in the reference range in the Warsaw Lab (1.03 MoM), while it was higher in the Moscow Lab (2.75 MoM).

In summary, median DPP-IV activity, as well as median DPP-IV MoM, were significantly elevated in all types of mucopolysaccharidoses (MPS), alpha-mannosidosis, sialidosis, gangliosidosis GM1, mucolipidosis type II/III (ML II/III), Wolman/CESD, multiple sulphatase deficiency (MSD) and ceroid lipofuscinosis type 2 (CLN2) as compared to controls.

The ROC curve analysis showed that DPP-IV activity discriminated some lysosomal diseases from healthy controls with 100% sensitivity and 100% specificity (area under ROC curve, i.e., AUC, 1.000, $p < 0.001$). In the Warsaw Lab (Supplementary Figures S2A and S2B and Supplementary Table S3) it concerned MPS I (cut-off ≥ 2.151 MoM), MPS II (cut-off ≥ 1.765 MoM), MPS IIIA (cut-off ≥1.609 MoM), MPS VI (cut-off ≥1.583 MoM), alpha-mannosidosis (cut-off ≥ 3.140 MoM), mucolipidosis II/III (cut-off ≥ 4.443 MoM), MSD (cut-off ≥ 1.623 MoM) and Wolman/CESD (cut-off ≥ 1.721 MoM). In the Moscow Lab (Supplementary Figure S3 and Supplementary Table S3) it concerned MPS IIIA (cut-off ≥ 3.955 MoM), MPS IIIB (cut-off ≥ 3.742 MoM) and mucolipidosis II/III (cut-off ≥ 3.624 MoM). Results obtained for other lysosomal diseases are summarized in Supplementary Figures S2 and S3.

Interestingly, DPP-IV activity was normal after bone marrow transplantation in an individual affected with MPS I (see Table 1 and Supplementary Table S1). DPP-IV was also in the reference range in both of his parents (obligate heterozygotes).

There was no significant difference in DPP-IV activity in patients affected with Gaucher disease (GD) types 1 and 2 (n = 1), who were not on enzyme replacement therapy (ERT), as well as in GD type 1 patients on ERT (MoM = 1.30, 1.67 and 1.39, respectively), see Table 1. DPP-IV activity in an obligate heterozygote for GD type 2 was within the reference range, see Supplementary Table S1.

In one patient with GD type 2 DPP-IV activity decreased with age and in one untreated patient with early onset Pompe disease DPP-IV increased (data not shown, available on request).

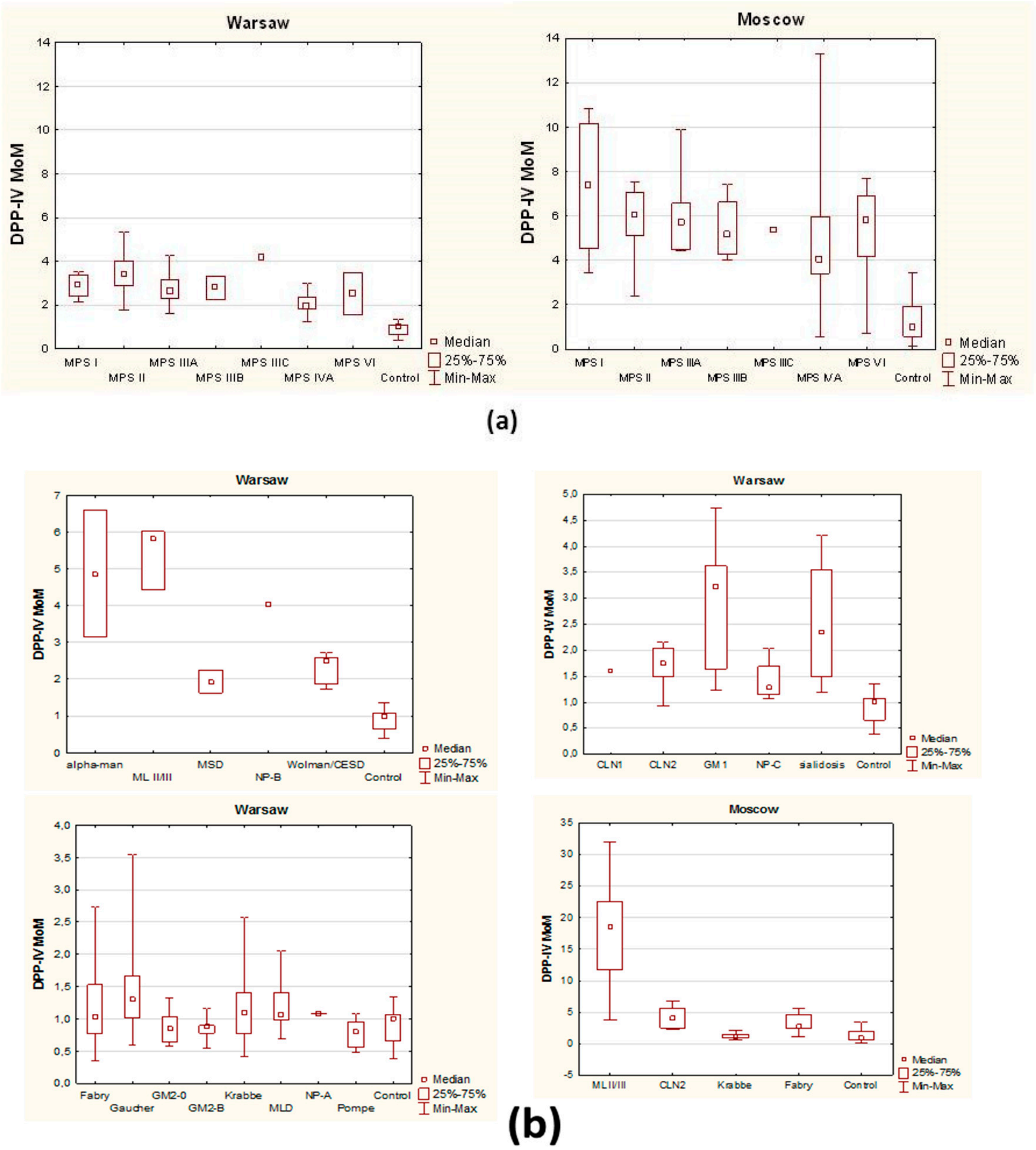

Figure 1. Dipeptidyl peptidase IV (DPP-IV) activity MoM (Multiple of the Median): (**a**) In patients with mucopolysaccharidoses (Warsaw and Moscow Labs); (**b**) in patients with other than mucopolysaccharidoses lysosomal diseases (Warsaw and Moscow Labs).

Table 1. DPP-IV activity in patients with lysosomal diseases presented as a multiple of the control group median (MoM).

**Disease**	**DPP-IV Activity—Multiple of Control's Median (MoM)**		**Results Abnormal/Total** [2]		**Number of Subjects; *n***	
	Lab Warsaw	Lab Moscow	Lab Warsaw	Lab Moscow	Lab Warsaw	Lab Moscow
**High DPP-IV activity**	data	data				
Mucolipidosis II/III	5.8	20.6	3/3	17/17	3	17
Alpha-mannosidosis	4.87	-	2/2	-	2	-
MPS t.IIIC[1]	4.2	-	1/1	-	1	-
Niemann–Pick t.B [1]	4.03	-	1/1	-	1	-

Gangliosidosis GM1	3.22	-	11/12	-	12	-
MPS t.II	3.16	6.06	12/12	8/10	12	10
**Elevated DPP-IV activity**						
MPS t.I	2.92	7.36	4/4	10/10	4	10
MPS t.I after BMT [1]	0.85	-	0/1	-	1	-
MPS t.IIIA	2.62	5.7	12/12	6/6	12	6
MPS t.IIIB	2.8	5.15	2/2	4/4	2	4
MPS t.IVA	1.93	4.1	4/5	10/14	5	14
MPS t.VI	2.53	5.61	2/2	10/11	2	11
sialidosis	2.35	-	3/4	-	4	-
Wolman/CESD	2.5	-	5/5	-	5	-
MSD	1.93	-	2/2	-	2	-
CLN2	1.75	5.37	19/21	12/19	21	19
**Slightly changed DPP-IV activity**						
Gaucher t.1	1.3	-	7/15	-	15	-
Gaucher t.1 on ERT	1.39	-	1/2	-	2	-
Gaucher t.2 [1]	1.67	-	1/1	-	1	-
CLN1 [1]	1.61	-	1/1	-	1	-
Niemann–Pick t.C	1.29	-	1/4	-	4	-
Niemann–Pick t.A	1.08	-	0/2	-	2	-
Fabry	1.03	2.75	4/10	4/10	10	10
Krabbe	1.09	1.17	8/25	0/10	25	10
Pompe	0.8	-	0/6	-	6	-
Gangliosidosis GM2-0	0.8	-	0/5	-	5	-
Gangliosidosis GM2-B	0.88	-	0/5	-	5	-
MLD	1.07	-	4/14	-	14	-

[1] DPP-IV analysis was performed on one person; [2] results were estimated as abnormal when they were higher than the maximal value of the reference range.

4. Discussion

The clinical symptom, which was displayed by all LSD patients with high DPP-IV was hepatomegaly. Interestingly, in untreated patients with Gaucher disease (GD), in whom the hepatomegaly is obvious, the DPP-IV activity was not elevated very much. Thus, it is proven that hepatomegaly in GD and aforementioned diseases results from different pathomechanisms. Enlargement of the liver or liver and spleen occurs in most lysosomal diseases. The severity of this symptom may range from a slight to a marked increase in liver (and spleen). Hepatomegaly may be accompanied by normal or impaired function of hepatocytes. For example, significant hepatomegaly occurs in patients with untreated Gaucher disease, in whom glucocerebroside storage takes place in macrophages (Browicz–Kupffer cells); however, the liver function remains normal. In the case of Niemann Pick A/B disease, the storage process affects macrophages and additionally the function of hepatocytes is disturbed. In MPS and glycoproteinoses, we note a moderate enlargement of liver and spleen volume without clinical significance. In these cases, the liver function is preserved or slightly impaired. In MPS accumulated GAGs disrupt mainly the connective tissue structure of the organ. In gangliosidoses (GM1 and GM2) there is hepatomegaly and damage of hepatocytes function.

It is worth noting that disturbances in the functionality of the immune system is a known and characteristic sign for these lysosomal diseases, in which DPP-IV activity was elevated. It has been shown that CD26/DPP-IV participates in the action of the immune

system: In maintaining lymphocyte composition and function, T cell activation and co-stimulation, memory T cell generation and thymic emigration patterns during immune-senescence; it is present in activated B cells, activated natural killer (NK) cells, eosinophils, macrophages and myeloid cells [17,18].

DPP-IV activity was elevated most in patients affected with alpha-mannosidosis or mucolipidosis II/III (ML II/III). Alpha-mannosidosis (OMIM 248500) is caused by the deficient activity of a lysosomal hydrolase, alpha-mannosidase (MAN2B1, EC 3.2.1.24), which is responsible for the degradation of N-linked oligosaccharides [19]. Mucolipidosis II/III (OMIM 252500, 252600 and 252605) is caused by a lack of active enzyme GlcNAc-1-phosphotransferase (GNPTAB, GNPTG, EC 2.7.8.17), which is involved in the phosphorylation step in the synthesis of the mannose 6-phosphate marker present on the lysosomal membrane. It can be speculated that a high amount of free mannose residues at the end of oligosaccharide chains of glycoproteins (in the case of alfa-mannosidosis patients) or at the cell surface (in the MLII/III cells) could have something in common with the increase of DPP-IV activity. Deficient or insufficient mannose 6 phosphorylation could result in disturbed immune response as indicated by Ikushima et al., who have identified the mannose 6-phosphate/insulin-like growth factor II receptor (M6P/IGFIIR) as a binding protein for CD26 and that mannose 6-phosphate (M6P) residues in the carbohydrate moiety of CD26 are critical for this binding. Activation of peripheral blood T cells results in the mannose 6 phosphorylation of CD26 [20].

At high concentrations in the cell, mannose is mostly catabolized to pyruvate, lactate and alanine, but not found in glycogen [21], which can be further associated with disturbed energetic metabolism leading to elevated DPP-IV activity. This may be also a cause of abnormal metabolism of amino acids and fatty acids observed in MPS patients and especially in patients with different types of MPS III [22]. Another explanation of high DPP-IV activity in MPS patients is the fact that glycosaminoglycans (GAGs, mucopolysaccharides), which are the main storage material, have been shown to bind strongly and selectively to several immune cytokines, leading to modulation of their bioactivity and/or tissue distribution [23]. GAGs are also involved in building the extracellular matrix (ECM), which is a potential candidate substrate for DPPIV, since limited proteolysis of ECM has been shown to play a role in cellular migration [24].

5. Conclusions

In conclusion, screening for the DPP-IV activity in plasma samples from patients with different lysosomal diseases (LSDs) revealed elevated activity of this enzyme, especially in individuals affected with mucolipidosis II/III, alpha-mannosidosis and mucopolysaccharidoses types III, II and I. While higher DPP-IV activities have already been described in MPS patients, finding that this enzyme is elevated also in some other LSD patients is a new observation. Thus, DPP-IV may serve as an economic, additional analysis in recognizing patients with some LSDs. Further studies on larger cohorts of patients are needed to estimate the value of DPP-IV as a potential biomarker. Fast and sensitive diagnosis is of peculiar importance for patients with diseases for which a treatment procedure is already available. Moreover, DPP-IV may become an object of basic research for better understanding LSDs.

Supplementary Materials: The following are available online at www.mdpi.com/2075-4418/11/2/320/s1. Supplementary Table S1 Raw data of DPP-IV activity in patients with lysosomal diseases. Supplementary Table S2A: DPP-IV activity (crude data) and DPP-IV activity expressed as MoM (multiple of the median) of the control group in various lysosomal diseases (Warsaw Lab.). Supplementary Table S2B: DPP-IV activity (crude data) and DPP-IV activity expressed as MoM (multiple of the median) of the control group in various lysosomal diseases (Moscow Lab.). Supplementary Table S3. Receiver-operator curve (ROC) analysis for the predictive accuracy of DPP4 activity in various lysosomal diseases. Supplementary Figure S1:

DPP-IV activity MoM in patients with lysosomal diseases. Supplementary Fig.S2A and S2B: The receiver operating characteristic (ROC) curves showing the predictive probabilities of DPP-IV activity MoM for lysosomal diseases (Warsaw Lab) Supplementary Fig.S3: The receiver operating characteristic (ROC) curves showing the predictive probabilities of DPP-IV activity MoM for lysosomal diseases (Moscow Lab)

Author Contributions: Conceptualization: A.Ł.; Methodology: A.Ł., M.B.-M., G.B., A.I., E.Z.; Validation: A.Ł., M.B.-M., G.B., A.I., E.Z.; Formal Analysis: A.Ł., M.B.-M., G.B., A.I., E.Z.; Investigation: A.Ł., M.B.-M., G.B., A.I., E.Z.; Resources: G.B., A.I., E.Z., A.G., H.M., T.K., K.S., J.W., J.K., A.T.-S., B.P.-S.; Data Curation: A.Ł., M.B.-M., G.B., A.I., E.Z.; Writing—Original Draft Preparation:, A.Ł.; Writing—Review & Editing: A.Ł., G.B., A.I., E.Z., M.B.-M., H.M., K.S., J.W., A.T.-S.; Visualization: M.B.-M., G.B.; Supervision: A.Ł, M.B.-M., E.Z. All authors reviewed and approved the final version of the manuscript. All authors have read and agreed to the final version of the manuscript.

Funding: This research received no external funding.

Institutional Review Board Statement: The study was conducted according to the guidelines of the Declaration of Helsinki. Ethical review and approval were waived for this study, due to the fact that plasma samples, surplus to the requirements for diagnosis, were collected with informed consent during the diagnostic procedures at the Institute of Psychiatry and Neurology, Dept. of Genetics (Warsaw, Poland) and in the Federal State Budgetary Institution, Research Centre for Medical Genetics (Moscow, Russia). Individual patient consent to participate was not sought since all procedures were in accordance with routine patient care for LSD screening and other diagnostic procedures. All experiments were performed in accordance with relevant guidelines and regulations.

Informed Consent Statement: Plasma samples used in this study were obtained from blood samples submitted to the Department of Genetics, Institute of Psychiatry and Neurology in Warsaw and to Research Centre for Medical Genetics in Moscow for routine LSD screening and other diagnostic procedures. Patients or their caregivers gave their informed consent to include their anonymized biological material in the scientific studies.

Data Availability Statement: The data presented in this study are available in this article or supplementary material section.

Acknowledgments: The authors would like to thank the physicians from hospitals in Poland and Russia for their co-operation.

Conflicts of Interest: The authors declare no conflict of interest.

References

1. Meikle, P.J.; Hopwood, J.J.; Clague, A.E.; Carey, W.F. Prevalence of Lysosomal Storage disorders. *JAMA* **1999**, *281*, 249–254.
2. Pinto, R.R.; Caseiro, C.; Lemos, M.; Lopes, L.; Fontes, A.; Ribeiro, H.; Pinto, E.; Silva, E.; Rocha, S.; Marcão, A.; et al. Prevalence of lysosomal storage diseases in Portugal. *Eur. J. Hum. Genet.* **2004**, *12*, 87–92.
3. Kingma, S.D.; Bodamer, O.A.; Wijburg, F.A. Epidemiology and diagnosis of lysosomal storage disorders; challenges of screening. *Best Pract. Res. Clin. Endocrinol. Metab.* **2015**, *29*, 145–157.
4. Poupetova, H.; Ledvinova, J.; Berna, L.; Dvorakova, L.; Kozich, V.; Elleder, M. The birth prevalence of lysosomal storage disorders in the Czech Republic: Comparison with data in different populations. *J. Inherit. Metab. Dis.* **2010**, *33*, 387–396.
5. Guo, Y.; He, W.; Boer, A.M.; Wevers, R.A.; De Bruijn, A.M.; Groener, J.E.M.; Hollak, C.E.M.; Aerts, J.M.F.G.; Galjaard, H.; Van Diggelen, O.P. Elevated Plasma Chitotriosidase Activity in Various Lysosomal Storage Disorders. *J. Inherit. Metab. Dis.* **1995**, *18*, 717–722.
6. Nilsson, O.; Svennerholm, L. Accumulation of glucosylceramide and glucosylsphingosine (psychosine) in cerebrum and cerebellum in infantile and juvenile Gaucher disease. *J. Neurochem.* **1982**, *39*, 709–718.
7. Rolfs, A.; Giese, A.-K.; Grittner, U.; Mascher, D.; Elstein, D.; Zimran, A.; Böttcher, T.; Lukas, J.; Hübner, R.; Gölnitz, U.; et al. Glucosylsphingosine is a highly sensitive and specific biomarker for primary diagnostic and follow-up monitoring in Gaucher disease in a non-Jewish, Caucasian cohort of Gaucher disease patients. *PLoS ONE* **2013**, *8*, e79732.
8. Smid, B.E.; van der Tol, L.; Biegstraaten, M.; Linthorst, G.E.; Hollak, C.E.; Poorthuis, B.J. Plasma globotriaosylsphingosine in relation to phenotypes of Fabry disease. *J. Med. Genet.* **2015**, *52*, 262–268.
9. Niemann, M.; Rolfs, A.; Störk, S.; Bijnens, B.; Breunig, F.; Beer, M.; Ertl, G.; Wanner, C.; Weidemann, F. Gene mutations versus clinically relevant phenotypes: Lyso-Gb3 defines Fabry disease. *Circ. Cardiovasc. Genet.* **2014**, *7*, 8–16.

10. Giese, A.-K.; Mascher, H.; Grittner, U.; Eichler, S.; Kramp, G.J.; Lukas, J.; Vruchte, D.T.; Al Eisa, N.; Cortina-Borja, M.; Porter, F.D.; et al. A novel, highly sensitive and specific biomarker for Niemann-Pick type C1 disease. *Orphanet J. Rare Dis.* **2015**, *10*, 78.
11. Aerts, J.M.F.G.; Kallemeijn, W.W.; Wegdam, W.; Ferraz, M.J.; Van Breemen, M.J.; Dekker, N.; Kramer, G.; Poorthuis, B.J.; Groener, J.E.M.; Cox-Brinkman, J.; et al. Biomarkers in the diagnosis of lysosomal storage disorders: Proteins, lipids, and inhibodies. *J. Inherit. Metab. Dis.* **2011**, *34*, 605–619.
12. Bobillo Lobato, J.; Jiménez Hidalgo, M.; Jiménez Jiménez, L.M. Biomarkers in Lysosomal Storage Diseases. *Diseases* **2016**, *4*, 40.
13. Beesley, C.E.; Young, E.P.; Finnegan, N.; Jackson, M.; Mills, K.; Vellodi, A.; Cleary, M.; Winchester, B.G. Discovery of a new biomarker for the mucopolysaccharidoses (MPS), dipeptidyl peptidase IV (DPP-IV.; CD26), by SELDI-TOF mass spectrometry. *Mol. Genet. Metab.* **2009**, *96*, 218–224.
14. Kurt, I.; Sertoglu, E.; Okur, I.; Tapan, S.; Uyanık, M.; Kayadıbı; H.; Ezgu, F.S.; Aydın, H.I.; Hasanoglu, A. Serum dipeptidyl peptidase-IV: A better screening test for early detection of mucopolysaccharidosis? *Clin. Chim. Acta.* **2014**, *431*, 250–254.
15. Hetmańczyk, K.; Bednarska-Makaruk, M.; Kierus, K.; Murawska-Izdebska, S.; Piekutowska-Abramczuk, D.; Pilch, B.; Tylki-Szymańska, A.; Ługowska, A. Monitoring of Dipeptidyl peptidase-IV (DPP-IV) Activity in Patients With Mucopolysaccharidoses Types I and II on Enzyme Replacement Therapy-Results of a Pilot Study. *Clin. Biochem.* **2016**, *49*, 458–462.
16. Mentlein, R. Dipeptidyl-peptidase IV (CD26)-role in the inactivation of regulatory peptides. *Regul. Pept.* **1999**, *85*, 9–24.
17. Klemann, C.; Wagner, L.; Stephan, M.; von Hörsten, S. Cut to the chase: A review of CD26/dipeptidyl peptidase-4's (DPP4) entanglement in the immune system. *Clin. Exp. Immunol.* **2016**, *185*, 1–21.
18. Wagner, L.; Klemann, C.; Stephan, M.; von Hörsten, S. Unravelling the immunological roles of dipeptidyl peptidase 4 (DPP4) activity and/or structure homologue (DASH) proteins. *Clin. Exp. Immunol.* **2016**, *184*, 265–283.
19. Beck, M.; Olsen, K.J.; Wraith, J.E.; Zeman, J.; Michalski, J.-C.; Saftig, P.; Fogh, J.; Malm, D. Natural History of Alpha Mannosidosis a Longitudinal Study. *Orphanet J. Rare Dis.* **2013**, *8*, 88.
20. Ikushima, H.; Munakata, Y.; Ishii, T.; Iwata, S.; Terashima, M.; Tanaka, H.; Schlossman, S.F.; Morimoto, C. Internalization of CD26 by Mannose 6-phosphate/insulin-like Growth Factor II Receptor Contributes to T Cell Activation. *Proc. Natl. Acad. Sci. USA* **2000**, *97*, 8439–8444.
21. Sharma, V.; Ichikawa, M.; Freeze, H.H. Mannose Metabolism: More Than Meets the Eye. *Biochem. Biophys. Res. Commun.* **2014**, *453*, 220–228.
22. Tebani, A.; Abily-Donval, L.; Schmitz-Afonso, I.; Héron, B.; Piraud, M.; Ausseil, J.; Zerimech, F.; Gonzalez, B.; Marret, S.; Afonso, C.; et al. Unveiling Metabolic Remodeling in Mucopolysaccharidosis Type III Through Integrative Metabolomics and Pathway Analysis. *J. Transl. Med.* **2018**, *16*, 248.
23. Gibbs, R.V. Cytokines and Glycosaminoglycans (GAGs). In *Glycobiology and Medicine*; Axford, J.S., Ed.; Kluwer Academic/Plenum Publishers: New York, NY, USA, 2003; pp. 125-143.
24. Vaday, G.V.; Lider, O. Extracellular matrix moieties, cytokines, and enzymes: Dynamic effects on immune cell behavior and inflammation. *J. Leukoc. Biol.* **2000**, *67*, 149–159.
